# Hexaaqua­manganese(II) bis­{[*N*-(3-meth­oxy-2-oxidobenzyl­idene)glycylglycinato]copper(II)} hexa­hydrate

**DOI:** 10.1107/S1600536810013061

**Published:** 2010-04-14

**Authors:** Long-Wei Lei, Yin-Zhi Jiang, Yang Zou

**Affiliations:** a Chemistry Department, Zhejiang Sci-Tech University, Hangzhou 310018, People’s Republic of China

## Abstract

The ligand *N*-(2-hydr­oxy-3-methoxy­benzyl­idene)glycylglycine (H_3_ *L*), a Schiff base derived from glycylglycine and 3-methoxy­salicylaldehyde, was used in the synthesis of a new organic–inorganic coordination complex, [Mn(H_2_O)_6_][Cu(C_12_H_11_N_2_O_5_)]_2_·6H_2_O. The Mn^II^ atom is located on an inversion center and is coordinated to six water mol­ecules in a slightly distorted octa­hedral geometry. The Cu^II^ atom is chelated by the tetra­dentate Schiff base ligand in a distorted CuN_2_O_2_ square-planar coordination. In the crystal structure, the complex [Mn(H_2_O)_6_]^2+^ cations and the [Cu*L*]^−^ anions are arranged in columns parallel to the *a* axis and are held together by O—H⋯O hydrogen bonding. Additional hydrogen bonds of the same type further link the columns into a three-dimensional network.

## Related literature

Transition metal complexes of salicylaldehyde–peptide- and salicylaldehyde–amino-acid-derived Schiff bases are suitable non-enzymatic models for pyridoxal amino acid systems, which are of considerable importance as key inter­mediates in metabolic reactions, see: Bkouche-Waksman *et al.* (1988 ▶); Wetmore *et al.* (2001 ▶); Zabinski & Toney (2001 ▶). For the preparation, structural characterization, spectroscopic and magnetic studies of Schiff base complexes derived from salicylaldehyde and amino acids, see: Ganguly *et al.* (2008 ▶) and references cited therein. For Schiff bases derived from simple peptides, see: Zou *et al.* (2003 ▶).
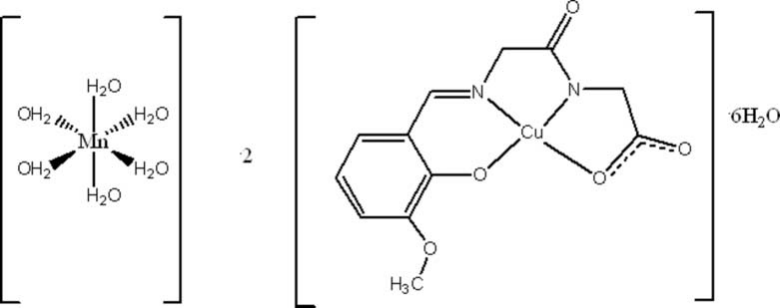


## Experimental

### 

#### Crystal data

[Mn(H_2_O)_6_][Cu(C_12_H_11_N_2_O_5_)]_2_·6H_2_O
*M* *_r_* = 924.67
Triclinic, 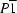

*a* = 6.712 (1) Å
*b* = 11.762 (2) Å
*c* = 12.092 (2) Å
α = 76.51 (1)°
β = 83.90 (1)°
γ = 80.37 (1)°
*V* = 912.9 (3) Å^3^
*Z* = 1
Mo *K*α radiation
μ = 1.59 mm^−1^
*T* = 293 K
0.3 × 0.2 × 0.2 mm

#### Data collection

Bruker SMART CCD diffractometer
Absorption correction: multi-scan (*SADABS*; Bruker, 2003 ▶) *T*_min_ = 0.690, *T*_max_ = 0.728
4571 measured reflections
3156 independent reflections
1625 reflections with *I* > 2σ(*I*)
*R*_int_ = 0.114

#### Refinement

*R*[*F* ^2^ > 2σ(*F* ^2^)] = 0.057
*wR*(*F* ^2^) = 0.129
*S* = 0.79
3156 reflections
246 parameters
114 restraints
H atoms treated by a mixture of independent and constrained refinement
Δρ_max_ = 0.74 e Å^−3^
Δρ_min_ = −0.56 e Å^−3^

### 

Data collection: *SMART* (Bruker, 2003 ▶); cell refinement: *SAINT* (Bruker, 2003 ▶); data reduction: *SAINT*; program(s) used to solve structure: *SHELXS97* (Sheldrick, 2008 ▶); program(s) used to refine structure: *SHELXL97* (Sheldrick, 2008 ▶); molecular graphics: *XP* in *SHELXTL* (Sheldrick, 2008 ▶) and *DIAMOND* (Brandenburg, 2000 ▶); software used to prepare material for publication: *SHELXTL*.

## Supplementary Material

Crystal structure: contains datablocks I, global. DOI: 10.1107/S1600536810013061/wm2319sup1.cif

Structure factors: contains datablocks I. DOI: 10.1107/S1600536810013061/wm2319Isup2.hkl

Additional supplementary materials:  crystallographic information; 3D view; checkCIF report

## Figures and Tables

**Table 1:** Selected bond lengths (Å)

Cu1—O2	1.873 (4)
Cu1—N2	1.887 (5)
Cu1—N1	1.905 (5)
Cu1—O4	1.979 (4)
Mn1—O6	2.161 (4)
Mn1—O7	2.173 (4)
Mn1—O8	2.212 (4)

**Table 2:** Hydrogen-bond geometry (Å, °)

*D*—H⋯*A*	*D*—H	H⋯*A*	*D*⋯*A*	*D*—H⋯*A*
O11*A*—H11*C*⋯O3^i^	0.77	1.93	2.689 (6)	172
O11*A*—H11*B*⋯O10^ii^	0.85	2.06	2.786 (7)	143
O10—H10*F*⋯O4	0.85	1.93	2.775 (6)	180
O10—H10*E*⋯O2	0.93 (7)	1.92 (7)	2.805 (7)	157 (6)
O9—H9*D*⋯O3	0.85	1.88	2.727 (6)	179
O9—H9*B*⋯O7	0.85	2.40	2.842 (6)	113
O8—H8*E*⋯O11*A*^iii^	0.85	2.12	2.786 (6)	135
O8—H8*D*⋯O10^iv^	0.85	2.17	2.795 (7)	130
O7—H7*C*⋯O9	0.85	2.35	2.842 (6)	117
O7—H7*B*⋯O5^v^	0.85	2.06	2.702 (6)	132
O6—H6*B*⋯O9^vi^	0.85	2.17	2.739 (5)	124

Symmetry codes: (i) 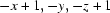; (ii) 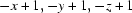; (iii) 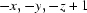; (iv) 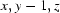; (v) 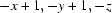; (vi) 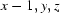.

**Fig. 1.**
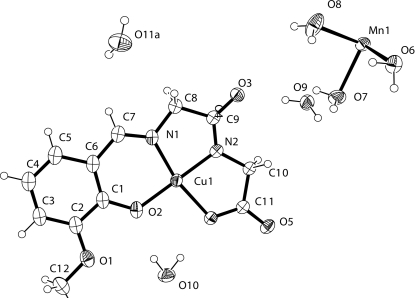
ORTEP plot of the asymmetric unit of complex (I) with the atom-numbering scheme (ellipsoids are drawn at the 40% probability level).

**Fig. 2.**
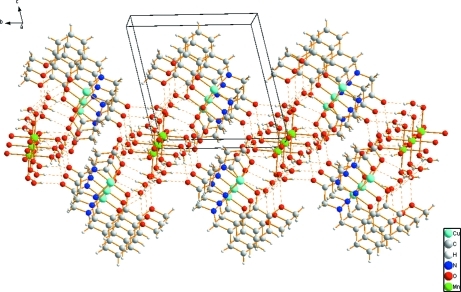
Representation of the hydrogen-bonded three-dimensional network (dashed lines) in the crystal structure of compound (I).

## supplementary crystallographic information

### Comment

Transition metal complexes of salicylaldehyde-peptides and salicylaldehyde-amino acid Schiff-bases are non-enzymatic models for pyridoxal-amino acid systems, which are of considerable importance as key intermediates in many metabolic reactions of amino acids catalyzed by enzymes (Zabinski *et al.*, 2001; Wetmore *et al.*, 2001; Bkouche-Waksman *et al.*,1988). Considerable effort has been devoted to the preparation, structural characterization, appropriate spectroscopic and magnetic studies of Schiff-base complexes derived from salicylaldehyde and amino acids and reduced salicylidene amino acids (Ganguly *et al.*, 2008), but little attention has been devoted to Schiff bases derived from simple peptides (Zou *et al.*, 2003). Herein, we report the structure study of [Mn(H_2_O)_6_][Cu(C_12_H_11_N_2_O_5_)]_2_^.^6H_2_O (H_3_*L*= Schiff base derived from glycylglycine and 3-methoxy-salicylaldehyde, C_12_H_14_N_2_O_5_).

The asymmetric unit of structure (I) consist of one-half of a [Mn(H_2_O)_6_]^2+^ cation (completed by crystallographic inversion symmetry), one [Cu*L*]^-^ anion and three water molecules (Fig. 1 and Table 1). The coordination environment of the Cu^II^ atoms is approximately square-planar. The Schiff-base ligand is deprotonated, thus acting as a triple negatively charged tetradentate *ONNO* chelate. It coordinates to the Cu^II^ atom via one phenolic oxygen atom (O2), one deprotonated amide nitrogen atom (N2), one imino nitrogen atom (N1) and one carboxylate oxygen atom (O4). The two Cu—N bond distances are 1.905 (5) Å (Cu1—N1) and 1.887 (5) Å (Cu1—N2). The two Cu—O bonds are 1.979 (4) (Cu1—O4) and 1.873 (4) Å (Cu1—O2). The phenyl ring [C1—C6] and the chelate ring [C1, C6, C7, N1, O2, Cu1] are almost coplanar with a small dihedral angle of 0.6°. The Mn^II^ center is octahedrally coordinated by the O atoms of six water molecules with Mn—O bond lengths in the range of 2.161 (4)-2.212 (4) Å.

Complex (I) shows an interesting stacking structure. The anions and cations of the complex form well-separated columns (Fig. 2) stacked along [100], held together by hydrogen bonding of the type O—H···O. The anion stacking is characterised by [Cu*L*]^-^ columns arranged in a zig-zag manner. The shortest Cu···Cu separation within a [Cu*L*]^-^ chain is 4.447 Å and the closest Cu···Cu separation between anionic chains is 9.312 Å. Additional O—H···O hydrogen bonds between the coordinated water molecules and uncoordinated water molecules further link the columns into a three-dimension network (Fig. 2, Table 1).

### Experimental

The Schiff base was prepared through the condensation of glycylglycine and 3-methoxy-salicylaldehyde. Glycylglycine (10 mmol) was dissolved and refluxed in absolute methanol (40 ml) containing LiOH^.^H_2_O (10 mmol). After cooling to room temperature, a solution of 3-methoxy-salicylaldehyde (10 mmol) in absolute methanol was added slowly under stirring for 10 min. Then Cu(NO_3_)_2_ (10 mmol) was added to the HLLi solution and the resulting solution was adjusted to pH = 9-11 by 1.0 mol/L NaOH solution. After stirring at room temperature for 30 min, the volume was reduced to ca. 5 ml *in vacuo*. Anhydrous ethanol was added to precipitate the product, which then was recrystallized in methanol solution. Na[Cu*L*]^.^2H_2_O (2 mmol) was dissolved in 10 ml water. Then MnCl_2_^.^4H_2_O (1 mmol) was added to the solution under stirring. The resulting crude product was precipitated. It was recrystallized in hot water 363 K and filtered. The filtrate was allowed to evaporate slowly at room temperature. After several days red to violet crystals suitable for X-ray diffraction were obtained.

### Refinement

The water H atoms in the complex were located in a difference Fourier map and were refined with a distance restraint of O—H = 0.85 Å and U_iso_(H) = 1.5U_eq_(O). All other H atoms were positioned geometrically and were constrained as riding atoms, with C—H distances of 0.93–0.97 Å and U_iso_(H) set to 1.2 or 1.5U_eq_(C) of the parent atom.

### Crystal data

**Table:**

[Mn(H_2_O)_6_][Cu(C_12_H_11_N_2_O_5_)]_2_·6H_2_O	*Z* = 1
*M**_r_* = 924.67	*F*(000) = 477
Triclinic, *P*1	*D*_x_ = 1.682 Mg m^−^^3^
Hall symbol: -P 1	Mo *K*α radiation, λ = 0.71073 Å
*a* = 6.712 (1) Å	Cell parameters from 1625 reflections
*b* = 11.762 (2) Å	θ = 1.7–25.0°
*c* = 12.092 (2) Å	µ = 1.59 mm^−^^1^
α = 76.51 (1)°	*T* = 293 K
β = 83.90 (1)°	Block, violet-red
γ = 80.37 (1)°	0.3 × 0.2 × 0.2 mm
*V* = 912.9 (3) Å^3^	

### Data collection

**Table:**

Bruker SMART CCD diffractometer	3156 independent reflections
Radiation source: fine-focus sealed tube	1625 reflections with *I* > 2σ(*I*)
graphite	*R*_int_ = 0.114
φ and ω scans	θ_max_ = 25.0°, θ_min_ = 1.7°
Absorption correction: multi-scan (*SADABS*; Bruker, 2003)	*h* = −7→7
*T*_min_ = 0.690, *T*_max_ = 0.728	*k* = −12→13
4571 measured reflections	*l* = −14→13

### Refinement

**Table:**

Refinement on *F*^2^	Primary atom site location: structure-invariant direct methods
Least-squares matrix: full	Secondary atom site location: difference Fourier map
*R*[*F*^2^ > 2σ(*F*^2^)] = 0.057	Hydrogen site location: inferred from neighbouring sites
*wR*(*F*^2^) = 0.129	H atoms treated by a mixture of independent and constrained refinement
*S* = 0.79	*w* = 1/[σ^2^(*F*_o_^2^) + (0.0082*P*)^2^] where *P* = (*F*_o_^2^ + 2*F*_c_^2^)/3
3156 reflections	(Δ/σ)_max_ < 0.001
246 parameters	Δρ_max_ = 0.74 e Å^−^^3^
114 restraints	Δρ_min_ = −0.56 e Å^−^^3^

### Special details

**Table:**

Geometry. All esds (except the esd in the dihedral angle between two l.s. planes) are estimated using the full covariance matrix. The cell esds are taken into account individually in the estimation of esds in distances, angles and torsion angles; correlations between esds in cell parameters are only used when they are defined by crystal symmetry. An approximate (isotropic) treatment of cell esds is used for estimating esds involving l.s. planes.
Refinement. Refinement of *F*^2^ against ALL reflections. The weighted *R*-factor wR and goodness of fit *S* are based on *F*^2^, conventional *R*-factors *R* are based on *F*, with *F* set to zero for negative *F*^2^. The threshold expression of *F*^2^ > σ(*F*^2^) is used only for calculating *R*-factors(gt) etc. and is not relevant to the choice of reflections for refinement. *R*-factors based on *F*^2^ are statistically about twice as large as those based on *F*, and *R*- factors based on ALL data will be even larger.

### Fractional atomic coordinates and isotropic or equivalent isotropic displacement parameters (Å^2^)

**Table:**

	*x*	*y*	*z*	*U*_iso_*/*U*_eq_	
Cu1	0.77185 (12)	0.49742 (7)	0.37778 (7)	0.0309 (3)	
C1	0.7114 (9)	0.5700 (6)	0.5897 (5)	0.0359 (10)	
C2	0.6755 (9)	0.6598 (7)	0.6508 (6)	0.0396 (11)	
C3	0.6653 (9)	0.6330 (7)	0.7685 (6)	0.0407 (12)	
H3	0.6421	0.6942	0.8071	0.049*	
C4	0.6886 (9)	0.5174 (6)	0.8304 (6)	0.0441 (13)	
H4	0.6799	0.5007	0.9097	0.053*	
C5	0.7244 (9)	0.4286 (7)	0.7730 (6)	0.0430 (13)	
H5	0.7406	0.3507	0.8143	0.052*	
C6	0.7379 (9)	0.4509 (6)	0.6520 (5)	0.0365 (11)	
C7	0.7766 (9)	0.3506 (6)	0.6003 (5)	0.0366 (12)	
H7	0.7891	0.2761	0.6487	0.044*	
C8	0.8340 (9)	0.2463 (5)	0.4483 (5)	0.0332 (12)	
H8A	0.7253	0.1997	0.4758	0.040*	
H8B	0.9599	0.1992	0.4746	0.040*	
C9	0.8469 (9)	0.2797 (6)	0.3194 (5)	0.0311 (11)	
C10	0.8264 (9)	0.4476 (5)	0.1580 (5)	0.0283 (11)	
H10A	0.7242	0.4223	0.1221	0.034*	
H10B	0.9579	0.4245	0.1214	0.034*	
C11	0.7866 (9)	0.5805 (6)	0.1443 (5)	0.0296 (11)	
C12	0.6232 (10)	0.8684 (6)	0.6400 (6)	0.0542 (19)	
H12A	0.4973	0.8690	0.6857	0.081*	
H12B	0.6206	0.9412	0.5837	0.081*	
H12C	0.7325	0.8602	0.6878	0.081*	
N1	0.7953 (7)	0.3554 (5)	0.4929 (4)	0.0317 (10)	
N2	0.8214 (7)	0.3923 (4)	0.2782 (4)	0.0285 (10)	
O1	0.6519 (6)	0.7718 (4)	0.5847 (4)	0.0457 (11)	
O2	0.7206 (6)	0.6012 (4)	0.4771 (4)	0.0378 (10)	
O3	0.8774 (6)	0.1989 (4)	0.2649 (4)	0.0394 (12)	
O4	0.7585 (6)	0.6211 (3)	0.2352 (3)	0.0315 (10)	
O5	0.7834 (6)	0.6436 (4)	0.0469 (4)	0.0384 (11)	
Mn1	0.5000	0.0000	0.0000	0.0327 (4)	
O6	0.1953 (6)	0.0535 (4)	−0.0541 (4)	0.0428 (13)	
H6C	0.1523	0.0503	0.0151	0.051*	
H6B	0.1485	0.1267	−0.0706	0.051*	
O7	0.5193 (6)	0.1775 (4)	0.0174 (4)	0.0477 (13)	
H7B	0.4022	0.2108	0.0358	0.057*	
H7C	0.5945	0.1606	0.0730	0.057*	
O8	0.3774 (6)	−0.0518 (4)	0.1787 (4)	0.0572 (15)	
H8D	0.4634	−0.0446	0.2224	0.069*	
H8E	0.2674	−0.0066	0.1889	0.069*	
O9	0.9213 (6)	0.2144 (4)	0.0353 (4)	0.0465 (13)	
H9D	0.9092	0.2092	0.1070	0.056*	
H9B	0.8259	0.2617	0.0008	0.056*	
O10	0.6740 (7)	0.8222 (4)	0.3246 (4)	0.0500 (14)	
H10F	0.6994	0.7607	0.2971	0.060*	
O11A	0.0288 (7)	0.0311 (4)	0.7410 (4)	0.0554 (15)	
H11B	0.1159	0.0700	0.7544	0.066*	
H11C	0.0589	−0.0354	0.7454	0.066*	
H10E	0.662 (10)	0.759 (6)	0.387 (6)	0.06 (2)*	

### Atomic displacement parameters (Å^2^)

**Table:**

	*U*^11^	*U*^22^	*U*^33^	*U*^12^	*U*^13^	*U*^23^
Cu1	0.0346 (5)	0.0328 (5)	0.0247 (5)	−0.0031 (4)	−0.0013 (4)	−0.0065 (4)
C1	0.0277 (19)	0.054 (2)	0.029 (2)	−0.0056 (18)	−0.0004 (18)	−0.0161 (19)
C2	0.029 (2)	0.057 (2)	0.036 (2)	−0.005 (2)	−0.0012 (19)	−0.020 (2)
C3	0.029 (2)	0.063 (3)	0.034 (2)	−0.005 (2)	−0.001 (2)	−0.022 (2)
C4	0.031 (2)	0.069 (3)	0.034 (2)	−0.007 (2)	0.000 (2)	−0.017 (2)
C5	0.031 (2)	0.066 (3)	0.032 (2)	−0.007 (2)	0.001 (2)	−0.011 (2)
C6	0.028 (2)	0.055 (2)	0.028 (2)	−0.0085 (19)	−0.0002 (18)	−0.0124 (19)
C7	0.030 (2)	0.050 (2)	0.030 (2)	−0.009 (2)	0.000 (2)	−0.006 (2)
C8	0.029 (2)	0.039 (2)	0.031 (2)	−0.006 (2)	−0.002 (2)	−0.005 (2)
C9	0.027 (2)	0.035 (2)	0.031 (2)	−0.006 (2)	−0.0011 (19)	−0.004 (2)
C10	0.024 (2)	0.032 (2)	0.027 (2)	−0.003 (2)	0.000 (2)	−0.005 (2)
C11	0.025 (2)	0.032 (2)	0.029 (2)	−0.002 (2)	0.0010 (19)	−0.0042 (19)
C12	0.053 (4)	0.061 (4)	0.058 (4)	−0.006 (3)	−0.004 (3)	−0.033 (4)
N1	0.028 (2)	0.041 (2)	0.027 (2)	−0.0088 (19)	−0.0008 (19)	−0.009 (2)
N2	0.024 (2)	0.031 (2)	0.029 (2)	−0.0047 (19)	−0.0009 (18)	−0.0025 (19)
O1	0.041 (2)	0.058 (3)	0.044 (2)	−0.005 (2)	−0.0023 (19)	−0.024 (2)
O2	0.035 (2)	0.050 (2)	0.030 (2)	−0.0042 (19)	−0.0017 (19)	−0.013 (2)
O3	0.046 (3)	0.035 (3)	0.036 (3)	−0.001 (2)	−0.001 (2)	−0.010 (2)
O4	0.034 (2)	0.030 (2)	0.029 (2)	−0.0028 (18)	0.0010 (18)	−0.0058 (18)
O5	0.039 (3)	0.039 (3)	0.031 (2)	0.000 (2)	0.000 (2)	−0.001 (2)
Mn1	0.0291 (9)	0.0317 (9)	0.0351 (9)	−0.0001 (7)	−0.0035 (7)	−0.0055 (7)
O6	0.029 (3)	0.052 (3)	0.047 (3)	0.005 (2)	−0.007 (2)	−0.016 (3)
O7	0.042 (3)	0.038 (3)	0.061 (3)	0.007 (2)	−0.016 (2)	−0.011 (3)
O8	0.036 (3)	0.084 (4)	0.041 (3)	0.004 (3)	−0.001 (2)	−0.001 (3)
O9	0.051 (3)	0.048 (3)	0.039 (3)	0.006 (2)	−0.011 (2)	−0.013 (3)
O10	0.064 (4)	0.034 (3)	0.052 (4)	−0.011 (3)	−0.006 (3)	−0.008 (3)
O11A	0.050 (3)	0.043 (3)	0.074 (4)	−0.010 (2)	0.003 (3)	−0.016 (3)

### Geometric parameters (Å, °)

**Table:**

Cu1—O2	1.873 (4)	C10—H10A	0.9700
Cu1—N2	1.887 (5)	C10—H10B	0.9700
Cu1—N1	1.905 (5)	C11—O5	1.237 (7)
Cu1—O4	1.979 (4)	C11—O4	1.282 (7)
C1—O2	1.323 (7)	C12—O1	1.424 (7)
C1—C2	1.400 (8)	C12—H12A	0.9600
C1—C6	1.418 (9)	C12—H12B	0.9600
C2—O1	1.366 (8)	C12—H12C	0.9600
C2—C3	1.382 (9)	Mn1—O6^i^	2.161 (4)
C3—C4	1.383 (9)	Mn1—O6	2.161 (4)
C3—H3	0.9300	Mn1—O7^i^	2.173 (4)
C4—C5	1.360 (8)	Mn1—O7	2.173 (4)
C4—H4	0.9300	Mn1—O8^i^	2.212 (4)
C5—C6	1.421 (9)	Mn1—O8	2.212 (4)
C5—H5	0.9300	O6—H6C	0.8500
C6—C7	1.434 (8)	O6—H6B	0.8500
C7—N1	1.280 (7)	O7—H7B	0.8500
C7—H7	0.9300	O7—H7C	0.8499
C8—N1	1.479 (7)	O8—H8D	0.8499
C8—C9	1.513 (8)	O8—H8E	0.8499
C8—H8A	0.9700	O9—H9D	0.8500
C8—H8B	0.9700	O9—H9B	0.8500
C9—O3	1.256 (7)	O10—H10F	0.8500
C9—N2	1.290 (7)	O10—H10E	0.93 (7)
C10—N2	1.447 (7)	O11A—H11B	0.8495
C10—C11	1.513 (8)	O11A—H11C	0.7651
			
O2—Cu1—N2	179.5 (2)	O4—C11—C10	117.6 (6)
O2—Cu1—N1	96.4 (2)	O1—C12—H12A	109.5
N2—Cu1—N1	83.4 (2)	O1—C12—H12B	109.5
O2—Cu1—O4	96.16 (18)	H12A—C12—H12B	109.5
N2—Cu1—O4	84.08 (19)	O1—C12—H12C	109.5
N1—Cu1—O4	167.49 (18)	H12A—C12—H12C	109.5
O2—C1—C2	118.0 (6)	H12B—C12—H12C	109.5
O2—C1—C6	123.8 (6)	C7—N1—C8	121.0 (6)
C2—C1—C6	118.2 (6)	C7—N1—Cu1	124.9 (5)
O1—C2—C3	124.6 (6)	C8—N1—Cu1	114.1 (4)
O1—C2—C1	114.6 (6)	C9—N2—C10	124.9 (5)
C3—C2—C1	120.7 (7)	C9—N2—Cu1	119.8 (5)
C2—C3—C4	121.7 (7)	C10—N2—Cu1	115.3 (4)
C2—C3—H3	119.2	C2—O1—C12	118.2 (5)
C4—C3—H3	119.2	C1—O2—Cu1	125.7 (4)
C5—C4—C3	118.6 (7)	C11—O4—Cu1	114.0 (4)
C5—C4—H4	120.7	O6^i^—Mn1—O6	180.0 (4)
C3—C4—H4	120.7	O6^i^—Mn1—O7^i^	91.61 (16)
C4—C5—C6	122.1 (7)	O6—Mn1—O7^i^	88.39 (16)
C4—C5—H5	118.9	O6^i^—Mn1—O7	88.39 (16)
C6—C5—H5	118.9	O6—Mn1—O7	91.61 (16)
C1—C6—C5	118.6 (6)	O7^i^—Mn1—O7	180.0 (2)
C1—C6—C7	124.0 (6)	O6^i^—Mn1—O8^i^	89.94 (16)
C5—C6—C7	117.4 (6)	O6—Mn1—O8^i^	90.06 (16)
N1—C7—C6	125.3 (7)	O7^i^—Mn1—O8^i^	92.27 (17)
N1—C7—H7	117.4	O7—Mn1—O8^i^	87.73 (17)
C6—C7—H7	117.4	O6^i^—Mn1—O8	90.06 (16)
N1—C8—C9	109.0 (5)	O6—Mn1—O8	89.94 (16)
N1—C8—H8A	109.9	O7^i^—Mn1—O8	87.73 (17)
C9—C8—H8A	109.9	O7—Mn1—O8	92.27 (17)
N1—C8—H8B	109.9	O8^i^—Mn1—O8	180.0 (4)
C9—C8—H8B	109.9	Mn1—O6—H6C	89.0
H8A—C8—H8B	108.3	Mn1—O6—H6B	119.0
O3—C9—N2	127.5 (6)	H6C—O6—H6B	90.0
O3—C9—C8	118.8 (5)	Mn1—O7—H7B	109.3
N2—C9—C8	113.7 (6)	Mn1—O7—H7C	99.5
N2—C10—C11	109.1 (5)	H7B—O7—H7C	110.9
N2—C10—H10A	109.9	Mn1—O8—H8D	108.6
C11—C10—H10A	109.9	Mn1—O8—H8E	109.3
N2—C10—H10B	109.9	H8D—O8—H8E	109.5
C11—C10—H10B	109.9	H9D—O9—H9B	112.9
H10A—C10—H10B	108.3	H10F—O10—H10E	74.7
O5—C11—O4	123.7 (6)	H11B—O11A—H11C	118.5
O5—C11—C10	118.7 (5)		

Symmetry codes: (i) −*x*+1, −*y*, −*z*.

### Hydrogen-bond geometry (Å, °)

**Table:**

*D*—H···*A*	*D*—H	H···*A*	*D*···*A*	*D*—H···*A*
O11A—H11C···O3^ii^	0.77	1.93	2.689 (6)	172
O11A—H11B···O10^iii^	0.85	2.06	2.786 (7)	143
O10—H10F···O4	0.85	1.93	2.775 (6)	180
O10—H10E···O2	0.93 (7)	1.92 (7)	2.805 (7)	157 (6)
O9—H9D···O3	0.85	1.88	2.727 (6)	179
O9—H9B···O7	0.85	2.40	2.842 (6)	113
O8—H8E···O11A^iv^	0.85	2.12	2.786 (6)	135
O8—H8D···O10^v^	0.85	2.17	2.795 (7)	130
O7—H7C···O9	0.85	2.35	2.842 (6)	117
O7—H7B···O5^vi^	0.85	2.06	2.702 (6)	132
O6—H6B···O9^vii^	0.85	2.17	2.739 (5)	124

Symmetry codes: (ii) −*x*+1, −*y*, −*z*+1; (iii) −*x*+1, −*y*+1, −*z*+1; (iv) −*x*, −*y*, −*z*+1; (v) *x*, *y*−1, *z*; (vi) −*x*+1, −*y*+1, −*z*; (vii) *x*−1, *y*, *z*.
